# Evaluation of an immunochromatographic assay for the detection of anti-hepatitis A virus IgM

**DOI:** 10.1186/1743-422X-7-164

**Published:** 2010-07-19

**Authors:** Hyeok-Jin Lee, Hye Sook Jeong, Byung-Ki Cho, Mi-Jeong Ji, Ji-Ha Kim, An-Na Lee, Kyoung-Ryul Lee, Doo-Sung Cheon

**Affiliations:** 1 Division of Enteric and Hepatitis Viruses, Center for Infectious Diseases, National Institute of Health, Korea Centers for Disease Control and Prevention, Seoul 122-701, South Korea; 2 Standard Diagnostics, Inc., Yongin 446-930, South Korea; 3 Seoul Medical Science Institutes, Seoul 140-809, South Korea

## Abstract

**Background:**

Hepatitis A virus (HAV) is a causative agent of acute hepatitis, which is transmitted by person-to-person contact and via the faecal-oral route. Acute HAV infection is usually confirmed by anti-HAV IgM detection. In order to detect anti-HAV IgM in the serum of patients infected with HAV, we developed a rapid assay based on immunochromatography (ICA) and evaluated the sensitivity of this assay by comparing it with a commercial microparticle enzyme immunoassay (MEIA) that is widely used for serological diagnosis.

**Results:**

The newly developed ICA showed 100% sensitivity and specificity when used to test 150 anti-HAV IgM-positive sera collected from infected patients and 75 negative sera from healthy subjects. Also, the sensitivity of ICA is about 10 times higher than MEIA used in this study by determining end point to detect independent on infected genotype of HAV. In addition, the ICA was able to detect 1 positive sample from among 50 sera from acute hepatitis patients that had tested negative for anti-HAV IgM using the MEIA.

**Conclusion:**

Conclusively, ICA for the detection of anti-HAV IgM will be very effective for rapid assay to apply clinical diagnosis and epidemiological investigation on epidemics due to the simplicity, rapidity and specificity.

## Background

Hepatitis A virus (HAV) is one of the common causative agents of acute hepatitis worldwide [1]. The clinical manifestation of HAV infection in humans can vary greatly, ranging from asymptomatic infection to fulminant hepatitis [2,3]. As a result of improvements in public sanitation and hygiene conditions, there has been a striking reduction in HAV endemicity in Western countries over the past few decades [4,5]. The shift from high to intermediate or low endemicity leads to a change in the age of individuals susceptible to hepatitis A, from children to adolescents or adults. In general, infection during childhood is asymptomatic, whereas infection in adults is often more severe. The standard diagnosis of acute hepatitis A is based on the detection of the immunoglobulin M (IgM) antibody to HAV (HAV-IgM) in patients who present with clinical features of hepatitis. Nevertheless, since many cases of hepatitis A are asymptomatic, HAV-IgM can be found in individuals who do not have clinical symptoms or biological abnormalities [6]. IgM antibodies directed against specific viral antigens can be detected due to nonspecific polyclonal activation of memory cells from a previous infection with an unrelated agent. Immune cells may become activated during viral infections or immune diseases [7-12]. Thus, in some cases, anti-HAV IgM detection could also correspond to immune reactivation.

The incubation period for HAV is from 2 to 6 weeks [13]. Anti-HAV IgM and IgG are detectable at the onset of symptoms [14], although virus can be detected in the blood and faeces sooner (10 to 12 days post-infection). Anti-HAV IgM continues to be detected in infected individuals for between 2 and 9 months post-infection [15], after which it declines. Therefore, anti-HAV IgM is useful for diagnosing acute or recent infection. Further, a diagnostic assay with high sensitivity for the detection of IgM is considered to be a valuable tool for the timely care of patients and for the control of infection during HAV epidemics in large population communities, including schools and military camps.

The anti-HAV IgG antibody increases gradually, reaching high levels during the convalescent phase [16] and remains for life, conferring immunity against reinfection. Secretory IgA is associated with intestinal resistance to many viral infections, as in the case of the polioviruses. The oral vaccine, obtained from attenuated viral strains, induces the production of this antibody and protects against enteric reinfection. In spite of this, some authors have shown that in cases of infection by HAV, intestinal immunity does not represent protection against this virus [14]. The routine laboratory diagnosis performed in cases of hepatitis A involves measuring the serum levels of intracellular hepatic enzymes (ALT and AST) as well as the detection of anti-HAV IgM antibodies in blood samples [17].

In this study, we developed an immunochromatographic assay for the detection of anti-HAV IgM and applied this to clinical specimens in order to determine the sensitivity and usefulness of this assay compared with a chemiluminescent-linked immunoassay that is widely used for the routine diagnosis of HAV infection.

## Results

### Sensitivity and specificity of the ICA compared with the MEIA

By using the ICA devised in this investigation, we were able detect anti-HAV IgM in 150 sera from patients with acute viral hepatitis (confirmed as recent HAV infections), who were infected by 1 of 3 different HAV genotypes. Further, anti-HAV IgM was not detected in 75 sera collected from normal subjects. In the testing of these sera, the ICA showed 100% sensitivity and specificity (Table 1). Among the 50 serum samples shown to be negative for anti-HAV IgM using the commercial MEIA, the presence of anti-HAV IgM was detected in 1 sample using the newly developed ICA.

**Table 1:** Comparison of ICA and MEIA (AxSYM HAVAB-M 2.0)

	ICA developed in this study		MEIA (AxSYM HAVAB-M 2.0)	
	
	+	-	+	-
HAV patients (genotypes IA, IIIA, and IB) (*n*^*a *^= 150)	150	0	150	0
Patients with hepatitis symptom (*n *= 50)	1	49	0	50
Normal subjects (*n *= 75)	0	75	0	75

^*a *^number of sample

### Comparison of assay sensitivity by determining the end-point detection limit

The ICA developed in this investigation could detect anti-HAV IgM from 10^-1 ^to 10^-4 ^dilutions of all tested serum; however, the "T" band of the ICA showed differing intensities with the 10^-4 ^dilutions according to the different HAV genotypes. In the serum from patients infected with HAV genotype IA, anti-HAV IgM was strongly detected in the 10^-4 ^serum dilution, whereas IgM was weakly detected in the 10^-4 ^dilutions of serum collected from patients infected with HAV genotypes IIIA and IB. Using the MEIA, we could detect IgM down to a dilution of 10^-3^, but could not detect it in the 10^-4 ^dilutions (Table 2).

**Table 2:** Detection of anti-HAV IgM in 10-fold serial dilutions of serum using ICA (SD Rapid test) and MEIA (AxSYM HAVAB-M 2.0)

**Genotype**	**ICA developed in this study**				**MEIA (COI)**			
		
	**10**^**-2**^	**10**^**-3**^	**10**^**-4**^	**10**^**-5**^	**10**^**-2**^	**10**^**-3**^	**10**^**-4**^	**10**^**-5**^
	
IA-1	++	++	++	-	+ (5.21)	+ (1.55)	- (0.46)	- (0.37)
IA-2	++	++	++	-	+ (5.14)	+ (1.78)	- (0.49)	- (0.36)
IIIA-1	++	++	+	-	+ (5.95)	+ (1.76)	- (0.48)	- (0.43)
IIIA-2	++	++	+	-	+ (4.72)	+ (1.35)	- (0.56)	- (0.31)
IB-1	++	++	+	-	+ (4.35)	+ (1.25)	- (0.37)	- (0.29)
IB-2	++	++	+	-	+ (4.58)	+ (1.01)	- (0.38)	- (0.29)

(ICA: ++ strong positive, + weak positive, - negative)

(MEIA: + positive, - negative, COI negative > 1 > positive)

## Discussion

In order to detect anti-HAV IgM in the serum of patients with HAV infection, we developed a diagnostic assay based on immunochromatography and evaluated the sensitivity of this assay using clinical specimens by comparing with an MEIA (AxSYM HAVAB-M 2.0). The sensitivity and specificity for anti-HAV IgM were both 100% in each of the 2 assays, as determined by the assay of 150 positive and 75 negative samples. However, among the 50 serum samples shown to be negative for anti-HAV IgM using the commercial MEIA, 1 sample was confirmed positive for anti-HAV IgM using the newly developed ICA. Although this specimen was considered to be negative based on optical density readings using the reagents supplied by the manufacturer, it has significant amount anti-HAV IgM less than cut off index (COI) (COI < 1.0) by MEIA. This suggests that the inconsistency between the 2 assays is attributable to their respective sensitivities.

Surprisingly, although we used only 5 μl of sera for the ICA, the sensitivity of the ICA was approximately 10 times higher than that of the MEIA used in this study based on a determination of the end-point detection limit independent of HAV genotype.

Because of the long incubation periods and late appearance of anti-IgM after HAV infection, timely medical care for patients with acute hepatitis and an effective control strategy during HAV-related epidemics in the community tend to be delayed [18]. Especially, earlier diagnosis based on higher sensitivity for close contact person without clinical symptoms among population with HAV epidemic can provide effective measure for determining strategy to stop spreading infections in community.

Although the MEIA using an automated system for general serological diagnosis can provide sensitive results compared other serologic assays, including the ELISA and rapid immunoassays reported previously, it involves the use of expensive equipment, and can be costly and relatively time-consuming when handling large sample sizes, as in an epidemic.

Therefore, many investigators have reported rapid serological assays for the detection of anti-HAV antibodies. One such assay is the dot immunogold filtration assay (DIGFA) for the detection of IgM antibody [19]. This assay does not require expensive laboratory equipment, and the sensitivity and specificity of this test have been shown to be 91.3% and 96.0%, respectively. However, both the sensitivity and specificity for anti-HAV IgM in DIGFA are lower than in the MEIA (AxSYM HAVAB-M 2.0).

In our study, the sensitivity of the ICA for anti-HAV IgM was demonstrated to be higher than that of the MEIA. In addition, performance of the ICA requires only a simple test kit without other detection equipment; therefore, the assay does not need expensive laboratory equipment or large amounts of space, as does DIGFA.

Synthetic peptides have also been used for the detection of HAV antibodies using biosensor technology based on surface plasmon resonance [20]. Using this system, a sensitivity range of 48% to 96% was achieved, the degree of sensitivity depending upon which peptide was used.

Even though this assay is in the developmental stage, biosensors can be more rapid than EIAs when they are fully automated and have potential clinical value; however, their expense may prove prohibitive [20]. In contrast, the ICA showed a high sensitivity comparable to MEIA; moreover, its use is not limited by relatively high costs.

However, although there was no significant difference in the specificities of the ICA and MEIA, the sensitivity of the ICA for HAV in sera was higher than that of the MEIA based on the end-point detection of antibody and the ability to detect anti-HAV IgM in samples that test negative using the MEIA. Owing to its rapidity and specificity, the ICA can be used as an effective assay for screening HAV infection. It takes less than 20 minutes to complete the assay, whereas the MEIA takes approximately 30 minutes. The shorter reaction time in the ICA does not influence its sensitivity and specificity. There is, however, a significant difference in the cost of the ICA and AxSYM HAVAB-M 2.0 systems. When assaying using the AxSYM HAVAB-M 2.0, expensive laboratory equipment, several regents, reagent vessels, matrix cells, and a sample cup are required. In addition, the AxSYM HAVAB-M 2.0 requires a certain amount of space for the necessary laboratory equipment. However, when assaying using the ICA developed in this study, we need only a simple test kit and a single reagent for diffusion. This means that the cost of detecting anti-HAV IgM using the ICA is considerably less than when using the AxSYM HAVAB-M 2.0.

## Conclusions

In conclusion, ICA can detect anti-HAV IgM effectively and use of this system without the help of extra apparatus enables to detect and screen HAV infection easily and rapidly, which can be widely used in an epidemiological survey.

## Methods

### Serum samples

All samples were obtained with informed consent and approval of the KCDC Institutional Review Board (IRB). One hundred and fifty serum samples were collected from patients with acute hepatitis who had HAV infection confirmed by serological diagnostic methods and molecular analysis for the determination of the infecting HAV genotype. Among the 150 sera, we identified 70 (46.7%) as being infected with genotype IA, 77 (51.3%) infected with IIIA, and 3 (2.0%) infected with IB (data not shown). As negative controls, 75 sera were also obtained from healthy individuals confirmed as having no recent HAV infection. Additionally, 50 serum samples were collected from patients with illness compatible with HAV infection but in whom the anti-HAV IgM had not been detected. We stored serum specimens at -70°C until use, and for the serological assays we used 5 μl of serum for the ICA and 150 μl of serum for the MEIA.

### Detection of anti-HAV IgM using the immunochromatographic assay

The ICA is designed for the detection of anti-HAV IgM in human serum or plasma. The ICA device has 2 precoated lines--"T" (an HAV IgM line coated with anti-human IgM) and "C" (a control line coated with goat anti-mouse IgG)--on the surface of the membrane. Purified HAV antigens and colloidal gold-conjugated HAV-specific monoclonal antibody were dried on a pad. The assay, based on immunochromatography, for the detection of anti-HAV IgM was performed strictly according to instructions. Briefly, 5 μl of serum or plasma drawn to black line into the square sample well marked "S" for sample inlet. Four drops of diluent containing 0.1 M phosphate buffer, 0.1% Tween 20, and 0.01% sodium azide was then introduced into the sample inlet and the test results were interpreted within 20 minutes.

The results were interpreted as follows. The control line should always appear in order to obtain a valid result for diagnosis--this indicates that the procedure has been performed correctly. If a purple "T" line is visible in the result window, a positive result is confirmed--this indicates that level of the anti-HAV IgM in the sample is above the detection limit. Otherwise, we confirm a negative result.

### Detection of anti-HAV IgM by the MEIA

AxSYM HAVAB-M 2.0 (Abbott Laboratory, USA) is based on microparticle enzyme immunoassay technology. The samples and all the AxSYM HAVAB-M 2.0 reagents required for one test were pipetted with the sampling probe into wells in the reaction vessel of the sampling centre. The reaction vessel was immediately transferred to the processing centre. Further pipetting was conducted in the processing centre using the processing probe. All steps were performed automatically and the diagnostic results were reported immediately. The AxSYM HAVAB-M 2.0 system includes the following: an antibody to human IgM (goat)-coated microparticles in TRIS buffer containing protein stabilizers (0.02% solids), human hepatitis A virus in phosphate buffer containing protein stabilizers (Titre ≥3,000, the virus was inactivated with formaldehyde), an antibody to hepatitis A virus (mouse, monoclonal), alkaline phosphatase conjugate in TRIS buffer containing protein stabilizers (0.9 μg/mL), specimen diluent containing sodium chloride in TRIS buffer, sodium azide, and antimicrobial agents.

### Determination of the detection limit of the ICA and the MEIA

We selected 3 sets of 6 sera with high titres of anti-HAV IgM, which were induced by infections with HAV of 3 different genotypes (IA, IB, IIIA) identified through molecular genotyping. The sera were diluted with phosphate-buffered saline (PBS) to 10-fold serial dilutions ranging from 10^-1 ^to 10^-5^. For a comparison of the detection limits, we performed both the ICA and MEIA using 5 serial dilutions of the 6 sera infected with HAV.

## Competing interests

The authors declare that they have no competing interests.

## Authors' contributions

HJL and HSJ performed much of the ICA and the MEIA, and drafted the manuscripts. BKC, MJJ, and JHK carried out development of the ICA. ANL and KRL tested anti-HAV IgM from patients with acute viral hepatitis, collected positive sera with anti-HAV IgM. DSC critically revised the manuscript and carried out the experiment design. All of the authors read and approved the final version of the manuscript.
